# Three-phase development of the health capital questionnaire: a mixed methods operationalization study

**DOI:** 10.3389/fpubh.2026.1829250

**Published:** 2026-05-18

**Authors:** Anna Schneider-Kamp, Anum Ilyas, Stine Lyngsø Beltoft, Sonja Wehberg, Søren Askegaard

**Affiliations:** 1 Department of Business and Management, University of Southern Denmark, Odense, Denmark; 2 Department of Mathematics and Computer Science, University of Southern Denmark, Odense, Denmark; 3 Department of Public Health, University of Southern Denmark, Odense, Denmark

**Keywords:** health capital, mixed-method approach, projective techniques, questionnaire development, systematic literature review

## Abstract

**Background:**

Health capital is emerging as a widely used conceptual framework offering an integrative sociocultural, economic, and behavioral resource-based perspective on individual health practices related to disease management and illness prevention. However, the full realization of its potential as an explanatory and predictive framework is currently somewhat constrained by the lack of an instrument that renders health capital observable.

**Objective:**

We aimed to operationalize the health capital conceptual framework through the development of a comprehensive questionnaire-based instrument for the assessment of the multiplicity of components of health capital and their interactions.

**Materials and methods:**

Drawing on the theoretical foundations of health capital, secondary evidence from a systematic literature review (*n* = 23 included articles), and primary evidence from expert focus groups (*n* = 23 experts) and projective techniques (*n* = 2,118 respondents), we conducted a three-phase development process. In this process, we distilled topics, identified dimensions of health capital, synthesized questionnaire items, and mapped them into the Health Capital Questionnaire (HCQ).

**Results:**

We developed the HCQ, comprising a total of 56 questions distributed over six sections, with translations into five languages: English, Italian, Romanian, Slovak, and Spanish. The mixed methods study employed in the development provided a solid basis for developing the questionnaire items for operationalizing the conceptual framework of health capital, demonstrating the viability of our proposed three-phase development process, which emphasizes validation-by-design in the absence of a clear external *post hoc* validation path.

**Conclusion:**

The Health Capital Questionnaire, in its current form, functions as a multi-purpose instrument capturing perceptions, behaviors, experiences, preferences, and attitudes. It allows for the assessment of sociocultural, economic, and behavioral aspects of disease management and illness prevention in a theoretically integrative, culturally adaptable, and contextually extensible manner.

## Introduction

The concept of health capital has seen a renaissance as an integrative resource-based framework following recent reconceptualization (1), which integrates social, economic, cultural, and symbolic health-related resources, including, but not limited to, relationships and social networks, financial means and assets, competencies and cultural backgrounds, and recognition and social status, respectively (2). In this sense, health capital can be understood as the accumulated set of health-related resources that individuals can draw on to maintain or improve their health and navigate formal and informal healthcare contexts. Health capital has been used as an analytic lens to study a wide variety of topics, including, but not limited to, sex differences in life expectancy (3), healthcare barriers across traditional and digital contexts (4), and the risk of rural exclusion from digital health services (5).

These and most other studies building on health capital rely on small-scale qualitative or case-based data collection methods such as interviews and comparative case analysis. Despite some early attempts to operationalize Schneider-Kamp’s health capital through proxy variables from a large-scale panel study in China (6) and a national representative survey in Israel (7), virtually all remaining research building on health capital remains conceptual in nature. For the full realization of health capital’s potential as an explanatory and predictive framework, an instrument that makes health capital observable at larger scales is warranted.

In this article, we aim to *systematically operationalize the health capital conceptual framework through the development of a comprehensive questionnaire-based instrument*.

Such a Health Capital Questionnaire (HCQ) ought to function as an instrument that allows for the faithful assessment of the multiplicity of components of health capital and captures their interactions. This article focuses on the first necessary step of making health capital observable; the development and subsequent external validation of measurement models linking questionnaire items to latent health capital constructs will constitute future research.

## Materials and methods

### Methodology

Health capital as a conceptual framework is not straightforwardly amenable to a standard questionnaire development approach, as health capital’s theoretical complexity precludes reducing all observations to a single numeric score (or even multiple ones). A reductionist approach inherently breaks down, as the value of health capital is not absolute but context-dependent (1). As an example, even a simple variable such as age is non-linearly associated with the endpoint of digital health adoption (8), with young adults being the most frequent users compared to children and older adults. Simultaneously, age is non-linearly associated with the endpoint of self-rated well-being, with middle-aged adults between 45 and 54 years of age reporting the lowest levels of subjective well-being (9).

Therefore, instead of aiming to measure health capital numerically once and for all, the HCQ was designed and developed to collect a set of observations relevant to health capital without presupposing any particular endpoint or the need for an associated measurement model. Although this allows data collected through the HCQ to be used for different endpoints, this design choice and development goal require a different approach to establishing validity (10) than typical statistical techniques used for *post hoc* external validation in psychometric scale development, such as exploratory or confirmatory factor analysis (11). We present a mixed methods approach grounded in a theoretical foundation and extensive, diverse empirical evidence from both secondary and primary sources. This validation-by-design approach aims to ensure both the relevance of each questionnaire item within a given category and the overall comprehensive coverage of the health capital conceptual framework.

**Figure 1:**
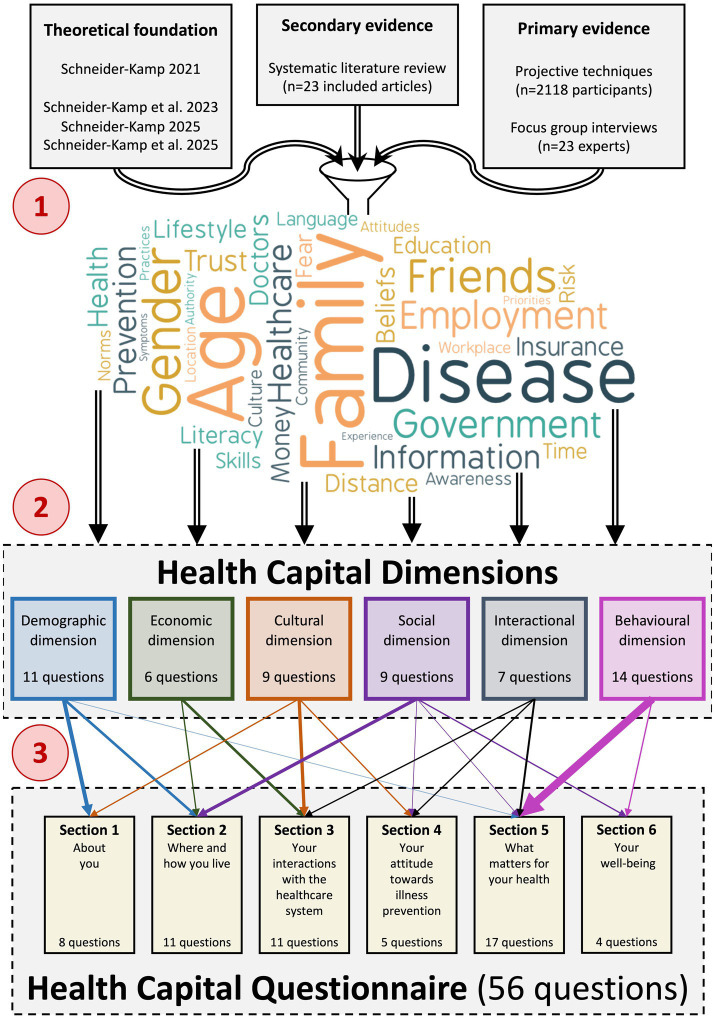
Inputs and artefacts of the three-phase questionnaire development process.

In the remainder of this section, we first introduce the setting of the development study and the different sources used in the questionnaire development process in the following subsection. Then, we describe our three-phase development process, followed by the *post hoc* testing and refinement process in the subsequent two subsections. The process is illustrated in Figure 1.

### Materials

This development study was embedded in the multi-country, multi-language, multi-center, and multi-cultural context of the EU-funded Horizon Europe research project Cancer Prevention at Work (12), which investigates how cancer prevention might be scaled through occupational health surveillance (13) and, among other objectives, aims to assess sociocultural and behavioral barriers and facilitators of prevention initiatives (14).

In the remainder of this subsection, we describe the sources used in our development process, ranging from the theoretical foundation to both secondary and primary empirical evidence. For each source, we provide a brief summary of its most pertinent results to facilitate an appraisal of its distinctive contributions and areas of convergence.

### Theoretical foundation

We define health capital as the *aggregate of the actual or potential resources possessed by a given agent that have the capacity to affect the agent’s position in the social field of health* (1). Different forms of health capital can be derived from one another through processes of conversion, with convertibility contingent on contextual factors such as healthcare financing and individual dispositions, including health information-seeking behavior.

Health capital is legitimized through a diverse set of structures, including medical authorities, market mechanisms, and, increasingly, online peers. In addition to classical Bourdieusian forms of capital such as social capital (social networks and norms), economic capital (financial means and valuable assets), and cultural capital (education and skills), health capital also encompasses personal antecedents that, together with these forms of capital, shape health practices and behaviors (15). Health capital not only acts as a facilitator of health encounters and self-care practices, but its absence acts as a barrier to effective healthcare service utilization.

In this respect, health capital differs from health literacy, which concerns individuals’ knowledge, motivation, and competencies to find, understand, evaluate, and apply health information (16), and from social determinants of health, which primarily are concerned with the structural conditions shaping health outcomes and inequalities (17). Rather than presuming a singular correct form of insight into health issues or focusing on structural conditions, health capital foregrounds and, more broadly, captures the uneven distribution of individuals’ health-related resources.

Health capital, therefore, functions comprehensively as both an explanatory and a predictive framework for disease management and illness prevention (2). One of its strengths is its ability to unveil health inequalities and its potential to suggest avenues for mitigation through a fine-grained perspective on which existing health-related resources might be leveraged and which additional ones might be needed.

### Secondary evidence from the literature (*n* = 23 included articles)

Taking onset in the prevention of infections related to cancers, where the impact of most prevention initiatives is hindered by less-than-optimal participation rates in screening, vaccination, and eradication programs, and noting that participation rates are modulated by various non-clinical barriers and facilitators, a systematic literature review (18) identified, aggregated, and validated social, cultural, economic, and personal factors influencing barriers and facilitators to cancer prevention.

Searching the Web of Science, PubMed, and Scopus databases for original articles published between 2013 and 2023, the review identified 353, 234, and 98 records, respectively, using a carefully curated keyword search. After duplicate removal, the resulting 685 records were assessed for eligibility, with a focus on infection type and prevention relevance. Articles addressing cost-effectiveness and psychological factors were excluded. The remaining 114 articles were subjected to full-text analysis, resulting in a final sample of 23 articles. Further details on the review methodology, including search strategies, eligibility criteria, and quality appraisal, are reported elsewhere (18).

The qualitative data synthesis found that social barriers included stigma, shame, weak family support, and patriarchal decision-making, while family encouragement, broader social support, and higher-status occupations functioned as facilitators. Cultural barriers included insufficient knowledge, awareness gaps, and fear of diagnosis or healthcare providers, while cultural facilitators included higher levels of education, positive screening norms, and language proficiency. Economic barriers included low income, high healthcare costs, and fear of income loss due to treatment side effects, while stable employment, insurance coverage, and subsidized services acted as facilitators. Regarding personal antecedents, barriers included reliance on spiritual healing, long distance from care services, and psychiatric side effects, while younger age, good health, and perceived susceptibility supported prevention and adherence to facilitated prevention.

### Primary evidence from expert focus groups (*n* = 23 experts)

In a focus group study conducted in November 2024, two focus groups with 8–12 healthcare workers as experts were recruited using purposive sampling (19) based on predefined eligibility criteria, with the aim of achieving a balanced sex distribution and broad coverage of age and educational backgrounds. These expert focus groups served a similar purpose to the recent use of the Delphi technique in questionnaire development for a multi-topic research framework (10), namely to provide expert grounding for the development process. In addition to group interviews and group discussions, the focus groups also incorporated word association and ordering projective techniques to elicit the experts’ underlying beliefs, associations, and cognitive pathways related to health-related behaviors and decision-making. Details on the data collection and coding process can be found elsewhere (20).

The thematic coding yielded four prevalent themes: *Lack of information and misinformation*, *distrust* and *refusal*, *work-related stress*, and *patient health education*. Regarding *lack of information*, the experts discussed patients being unaware of the services available to them and of the importance of prevention. In relation to *misinformation*, the experts discussed vaccination myths, the spread of misinformation through the internet, and the improper use of medication due to preconceived attitudes. Regarding *distrust* and ensuing *refusal*the experts noted that diffuse patterns of distrust toward authorities directly impacted the interaction between patients and healthcare workers and often led to refusal, that is, non-compliance with professional recommendations. Regarding *work-related stress*, this was often attributed to night shifts, working in complex environments, and dealing with challenging clients, as well as friction with management. Regarding *patient health education*, the experts considered it a crucial factor for the effectiveness of healthcare and discussed pathways for increasing health knowledge in the broader population through, for example, rural health education initiatives.

### Primary evidence from projective techniques (*n* = 2,118 respondents)

To assess the self-rated prioritization of health-related resources, a projective technique with a drawing task—the 12 Circles Drawing Technique (12-CDT) (21)—was developed. Projective techniques have long been a cornerstone of social and behavioral science research, enabling the exploration of participants’ implicit thoughts, feelings, and perceptions (22, 23). By engaging participants in creative tasks, these methods bypass the constraints of direct questioning, eliciting less rationalized and more spontaneous responses compared to traditional qualitative methods such as interviews and focus groups. The expectation is that responses obtained through projective techniques will be less influenced by the impact of reflexive response biases compared to conventional qualitative methods (24).

The 12-CDT was designed with cultural flexibility in mind, providing a visual and interactive means for participants to articulate their perceptions of health-related resources. To ensure that the technique was comprehensible to both respondents and administrators, the 12-CDT was designed to be intuitive and user-friendly, accommodating different cognitive and cultural processing styles. The 12-CDT presented 12 statements derived from the health capital framework, organized around the overarching question “How important are each of these to your health?” applied to the distinction between social, cultural, and economic forms of capital, as well as personal antecedents.

**Figure 2:**
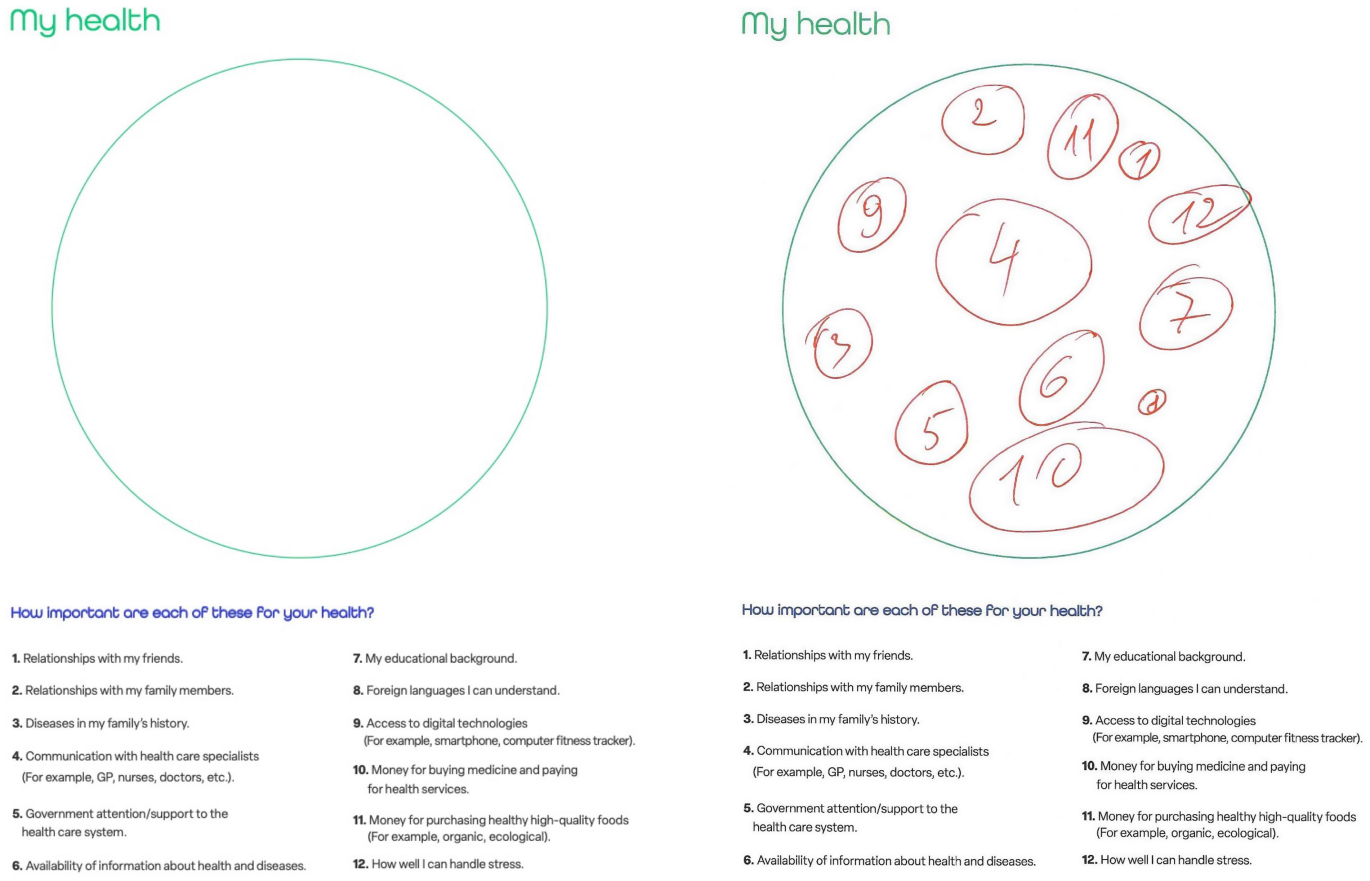
An unfilled (left) and a filled (right) circle diagram of the 12-CDT (34).

Respondents engaged with the 12-CDT by using a large circle that served as a projective space in which they could indicate their level of self-rated importance for each of the 12 statements describing the associated health-related resources. Respondents were instructed to draw larger circles for statements that were more significant to them and smaller circles for those that were less significant. Each circle was marked with the number of the corresponding statement. Figure 2 illustrates this by showcasing both an unfilled and a filled circle diagram of the 12-CDT.

A total of 2,118 respondents completed the 12-CDT, of whom 68.4% identified as female, 22.8% identified as male, and 3.4% chose not to disclose their sex. Across 11 institutions implementing prevention initiatives in four countries (Italy, Romania, Slovakia, and Spain), a clear majority of participants indicated that “Relationships with my family members” was their number one health priority among the other options presented. By a considerable margin, “How well I can handle stress” was ranked second, followed by “Diseases in my family’s history” in third place. At the other end of the spectrum, “Access to digital technologies” and “Foreign languages I can understand” were prioritized the least.

“Relationships with my family members” was most strongly correlated with “Relationships with my friends,” with a correlation score of 0.67. The highest correlation observed in the data was between “Foreign languages I can understand” and “Access to digital technologies” (0.83), followed by the correlations between “Availability of information on health and diseases” and both “Access to digital technologies” and “Foreign languages,” with correlation scores of 0.83 in each case. Although “How well I can handle stress” was ranked second most frequently as a top priority after “Relationships with my family members,” there was a relatively low correlation (0.33) between these two factors.

Self-rated priorities were relatively stable when comparing female and male respondents. Among younger respondents (aged <35), the distribution of rankings did not differ substantially, with one notable exception: “Money for buying medicine and paying for health services” was ranked as a top priority considerably less frequently than in the overall respondent group.

### Three-phase development process

To systematically leverage, integrate, and triangulate the different sources, we employed a three-phase development process:

#### Topic distillation

First, we identified topics from the theoretical foundation, as well as the sources of secondary and primary evidence, by systematically tabulating topics mentioned with respect to their potential impacts on disease management and illness prevention. We clustered the topics into categories and subsequently integrated them into higher-level categories or dimensions in a process reminiscent of open coding followed by theory-aware axial and selective coding (25). We halted the iterative integration process when we judged that further integration would lead to overgeneralization.

#### Questionnaire item synthesis

Then, we systematically addressed each dimension through the iterative synthesis (26) of questionnaire items (i.e., pairs of questions and answer options). For each questionnaire item generated, we assessed the degree to which it overlapped with existing items in the same category. In the case of significant overlap, we either discarded the new item, discarded the old item, or merged the two items. The answer options were carefully curated by revisiting all sources supporting the respective questionnaire item.

#### Questionnaire mapping

Finally, we rearranged the questionnaire items to improve their fit with the anticipated cognitive world of respondents (27). We pursued a respondent-centered design by organizing the questions according to a logical flow of everyday themes, rather than the theory- and data-driven top-level categories developed during topic distillation. We sectioned the questionnaire thematically and generated headings and brief explanations.

This process, which grounds the development of the HCQ firmly in diverse sources, provides a reproducible framework that is both theory- and data-driven. The inherent support of individual questionnaire items in one or more of these sources forms the backbone of this validation-by-design approach to questionnaire development.

### *Post hoc* testing and refinement

We pre-tested early versions of the HCQ on several small-scale convenience samples of adults of varying age, sex, and educational backgrounds. Based on completion time and self-reported levels and sources of frustration, we cautiously refined the order and wording of some of our questionnaire items, reducing response time per respondent and minimizing frustration.

### Ethics approval

This questionnaire development study, conducted under the Cancer Prevention at Work project, adheres to the Declaration of Helsinki and received ethical approval from the University of Bologna on 29 February 2024, as well as from the relevant local ethics committees at each site of primary data collection. Specifically, approvals were obtained from the following bodies: Comitato di Bioetica Alma Mater Studiorum Università di Bologna (29 February 2024), Comitato di Bioetica d’Ateneo Università di Torino (19 March 2024), Comisia de Etică a Cercetării din Spitalul Colentina (26 February 2024), the Timisoara Municipal Emergency Clinical Hospital (19 January 2024), Etická Komisia FNsP F.D. Roosevelta Banská Bystrica (27 February 2024), the Advisory Group of the Regional Hygienist RAPH BB (5 March 2024), Nezávislá Etická Komisia Banskobystrického (21 May 2024), and Comité de Ética de la Investigación del Principado de Asturias (14 December 2023). The data processing was approved by the Danish Data Protection Agency through the Research and Innovation Organization of the University of Southern Denmark, under journal number 12.277 on 27 June 2024. Informed consent was obtained from all participants.

## Results

The results of the three phases of the questionnaire development process are illustrated alongside the three-phase development process in Figure 1. At the top, we present the theoretical foundation and the secondary and primary sources of evidence, which, in Phase 1, gave rise to 37 topics, illustrated with a word cloud. To arrive at these topics, and drawing on primary evidence such as the 12-CDT data, we ranked and applied thresholds to potential topic candidates. The 37 topics were clustered and integrated into six top-level categories, taking into account the distinction between social, economic, and cultural forms of health capital, as well as personal antecedents (15). We identified these top-level categories as the *demographic*, *economic*, *cultural*, *social*, *interactional*, and *behavioral* dimensions of health capital, where the demographic and behavioral dimensions resulted from two distinct clusters adjacent to personal antecedents, while the interactional dimension emerged from a cluster at the intersection of cultural and social forms of capital.

For each of these dimensions, in Phase 2, we generated questionnaire items, discarding or merging them as appropriate during the process. For example, we discarded a question on how often healthcare costs prevent respondents from obtaining necessary care, as it overlapped with items addressing whether their healthcare needs are met and whether they can afford prescribed treatments. We also merged a question on the potential migration status of the respondent’s family with one on the migration status of their parents into a single question on the respondent’s migration status, with extended answer options. This iterative process finally yielded a total of 56 questions, with a minimum of six and a maximum of 14 questions per dimension. We mapped these 56 questions into six sections, with a minimum of four and a maximum of 17 questions per section.

In the following six subsections, we describe the topics, their integration, and the synthesis of questionnaire items for each dimension, before concluding with a short description of and reference to the rearranged HCQ.

### Demographic dimension: personal characteristics and geographic placement

Theoretically, the demographic dimension drew upon the notions that an individual’s health depends “on both the health and the health attitudes of family members” (1), that “the capacity to fully care for themselves” depends on age (15), that sex interacts with “identity disparities and sociocultural and economic disparities,” and that rural settings are often characterized “by an intersection of lower incomes, lower education, and higher social cohesion” (2). The secondary data further suggested that cultural embeddedness, cohabitation, and travel time and distance to healthcare facilities play key roles (18).

The expert focus groups discussed the risks of side effects from prevention activities, such as vaccination and screening. The 12-CDT data overwhelmingly supported the importance of family, with “Relationships with my family members” emerging as the top-ranked self-rated health priority across national, cultural, and occupational boundaries. The prevalence of “Diseases in my family’s history” also persisted across different contexts.

Integrated, the sources jointly suggested topics such as sex, age, disease, health, culture, family, risk, location, distance, and time as constituent elements of the demographic dimension. The iterative synthesis of questionnaire items for this dimension yielded a total of 11 pairs of questions and answer options, which are presented in the refined version in Table 1.

**Table 1:** Questionnaire items synthesized for the demographic dimension of health capital.

Questionnaire item	Answer options
Sex	Male Female Other/Undisclosed
Age (years)	18–24 25–34 35–44 45–54 55–64 65 or over Prefer not to disclose
Height (cm)	__________________________________
Weight (kg)	__________________________________
Ethnicity	American Indian or Alaska Native: A person having origins in any of the original peoples of North and South America (including Central America) and who maintains tribal affiliation or community attachment. Asian: A person having origins in any of the original peoples of the Far East, Southeast Asia, or the Indian subcontinent, including Cambodia, China, India, Japan, Korea, Malaysia, Pakistan, the Philippines Islands, Thailand, and Vietnam. Black or African American: A person having origins in any of the Black racial groups of Africa. Hispanic or Latino: A person of Cuban, Mexican, Puerto Rican, South or Central American, or other Spanish culture or origin, regardless of ethnicity. The term “Spanish origin” can be used in addition to “Hispanic or Latino.” Native Hawaiian or Other Pacific Islander: A person having origins in any of the original peoples of Hawaii, Guam, Samoa, or other Pacific Islands. White: A person having origins in any of the original peoples of Europe, the Middle East, or North Africa. Other, specify ______
What describes your background?	I was born in this country, and my family has been here for several generations. I was born in this country, but my parents moved here from somewhere else. I was born in a different country, but my family moved to this country when I was a child. I moved to this country as an adult. Prefer not to disclose.
Marital status	Single Married Cohabitating with a partner (not married) Divorced/separated Widowed Prefer not to answer
Are there histories of illnesses and health issues in your family that make you worry about your own health?	Yes, and it makes me worry about my health. Yes, but I do not worry about it. I am not aware of any such history.
What type of area do you currently live in?	Urban (cities, larger towns) Rural (smaller towns, villages, countryside)
Do you feel safe outside your home in your local area?	Definitely yes Probably yes Unsure Probably not Definitely not
How long does it take to reach the nearest healthcare facility (family doctor, hospital, clinic) from your home?	Less than 30 min away 30 min to 1 h More than 1 h away

### Economic dimension: financial situation and decision-making

Theoretically, the economic dimension drew upon the notions that one would “expect a higher degree of convertibility” of monetary resources into other health-related resources in insurance-based healthcare systems compared to those built on the idea of “virtually free” universal healthcare (1) and that family support (or the lack thereof) is a key factor in the management of both acute and chronic conditions (15). The secondary data further suggested that “lack of trust in medical doctors and medical care services” presents a major barrier, while governmental prioritization of prevention initiatives serves as a major facilitator (18).

The expert focus groups discussed how doctors are increasingly viewed as service providers rather than experts with medical authority—a point well established in prior research (28)—and how cost prevents economically less advantaged individuals from filling prescriptions when their focus is on providing necessities for their families. The 12-CDT data likewise suggested that “Money for buying medicine and paying for health services” and “Money for buying high-quality foods” are concerns for certain population segments.

Integrated, the sources jointly suggested topics such as money, healthcare, employment, doctors, insurance, government, priorities, and family as constituent elements of the economic dimension. The iterative synthesis of questionnaire items for this dimension yielded a total of six pairs of questions and answer options, which are presented in the refined version in Table 2.

**Table 2:** Questionnaire items synthesized for the economic dimension of health capital.

Questionnaire item	Answer options
What is your role in your household when it comes to financial support? (Select all that apply)	◦ I am the sole provider. ◦ Financial responsibilities are shared. ◦ I provide for others. ◦ Others provide for me.
How often do you worry about being able to afford essentials such as rent, food, and/or utilities?	◦ Never ◦ Rarely ◦ Sometimes ◦ Often ◦ Always
Does the public healthcare system meet your needs when you require medical care?	◦ Never ◦ Rarely ◦ Sometimes ◦ Often ◦ Always
Does your family doctor (general practitioner) meet your needs and expectations during visits?	◦ Never ◦ Rarely ◦ Sometimes ◦ Often ◦ Always
How often do you skip or reduce the use of prescribed medication due to cost?	◦ Never ◦ Rarely ◦ Sometimes ◦ Often ◦ Always
How often do financial constraints influence your ability to make healthier choices, such as healthy eating and physical exercise?	◦ Never ◦ Rarely ◦ Sometimes ◦ Often ◦ Always

### Cultural dimension: health literacy and health information seeking

Theoretically, the cultural dimension drew upon the notion that basic health literacy provides the basis for self-efficacy and that the “broad availability of online medical information” and medical authority serve as legitimation mechanisms for health capital (1). It further considered that trust and education enable communication, while language mismatch hinders it (15), and that the relationship between cultural “resources and health beliefs” is complex (2). The secondary data confirmed the important role of health beliefs and further found that awareness is a facilitator of preventive practices (18).

The expert focus groups revealed that, while doctors play an important role in patient health education, governmental support is crucial. The 12-CDT data were less conclusive, with both “Government attention/support to the healthcare system” and “Communication with healthcare specialists” rarely emerging as top priorities for respondents.

Integrated, the sources jointly suggested topics such as education, literacy, information, language, trust, government, authority, beliefs, awareness, prevention, and doctors as constituent elements of the cultural dimension. The iterative synthesis of questionnaire items for this dimension yielded a total of nine pairs of questions and answer options, which are presented in the refined version in Table 3.

**Table 3:** Questionnaire items synthesized for the cultural dimension of health capital.

Questionnaire item	Answer options
Education	◦ None ◦ Primary ◦ Secondary (below university level) ◦ Higher (university or above) ◦ Prefer not to answer
How many foreign languages do you speak?	◦ I do not speak any foreign language. ◦ 1 ◦ 2 ◦ 3 ◦ 4 ◦ 5 or more
Do you understand the health information provided by your family doctor or other healthcare professionals (e.g., medical specialists and nurses)?	◦ Definitely yes ◦ Probably yes ◦ Unsure ◦ Probably not ◦ Definitely not
Do you understand health-related information you find on your own (e.g., online or in the media)?	◦ Definitely yes ◦ Probably yes ◦ Unsure ◦ Probably not ◦ Definitely not
From whom/where do you get reliable health-related information? (Select all that apply)	◦ My healthcare provider ◦ Websites, apps, and social media ◦ Family or friends ◦ Books and journals ◦ My religious leader or faith group ◦ Other ____________
Do you trust health-related government guidelines for you and your family?	◦ Never ◦ Rarely ◦ Sometimes ◦ Often ◦ Always
What would strengthen your trust in government health policies or programs? (Select all that apply)	◦ Transparency in planning and decision-making. ◦ Public involvement in planning and decision-making. ◦ Regular and consistent communication. ◦ Policies based on science and evidence. ◦ Other _______________ ◦ I already fully trust them.
◦ Do you believe screening can help detect and therefore treat cancer?	◦ Definitely yes ◦ Probably yes ◦ Unsure ◦ Probably not ◦ Definitely not
Do you believe vaccines can effectively prevent cancers caused by infections (such as human papillomavirus and cervical cancer, *Helicobacter pylori* and stomach cancer, and hepatitis C virus and liver cancer)?	◦ Definitely yes ◦ Probably yes ◦ Unsure ◦ Probably not ◦ Definitely not

### Social dimension: social support networks and peer impact

Theoretically, the social dimension drew upon the notions that social networks and their norms directly affect health-related practices and health outcomes (1), that the presence or absence of family members plays a critical role in both chronic and acute care pathways (15), and that online communities have the potential to both empower patients and disrupt healthcare (2). The secondary data further suggested that social stigma is an often overlooked factor in questions regarding prevention participation (18).

The expert focus groups discussed the important role of family in ensuring mental well-being. The 12-CDT data once again confirmed the importance of “Relationships with my family members” and “Relationships with my friends,” suggesting a significant impact on lifestyle and health-related decision-making.

Integrated, the sources jointly suggested topics such as family, friends, community, norms, lifestyle, prevention, and health as constituent elements of the social dimension. The iterative synthesis of questionnaire items for this dimension yielded a total of nine pairs of questions and answer options, which are presented in the refined version in Table 4.

**Table 4:** Questionnaire items synthesized for the social dimension of health capital.

Questionnaire item	Answer options
How many adult (18 + years) household members do you have?	___________
How many of your household members are below 18 years?	___________
What is your role in your household when it comes to making decisions about healthy eating? (Select all that apply)	◦ I make my own decisions. ◦ Decisions are shared. ◦ I make decisions for others. ◦ Others decide for me.
What is your role in your household when it comes to making decisions about physical exercise? (Select all that apply)	◦ I make my own decisions. ◦ Decisions are shared. ◦ I make decisions for others. ◦ Others decide for me.
What is your role in your household when it comes to making decisions about contact with GP/health professionals? (Select all that apply)	◦ I make my own decisions. ◦ I make decisions for others. ◦ Decisions are shared. ◦ Others decide for me.
Do people you know usually participate in health checks or prevention programs such as vaccination, cancer screening, or tests for infections?	◦ Never ◦ Rarely ◦ Sometimes ◦ Often ◦ Always
How have the habits of your family made it harder for you to stay healthy? (Select all that apply)	◦ Expectations around eating or dieting. ◦ Cultural or religious practices. ◦ Social habits such as drinking or smoking. ◦ Not at all.
How do your friends, colleagues, and other people you know influence your health-related decisions e.g., lifestyle, vaccination, and screening? (Select all that apply)	◦ They encourage me to make healthy decisions. ◦ They offer me support. ◦ They judge me for my health-related decisions. ◦ They have no influence on my decisions.
When you are unwell, who in your life do you rely on for support such as transportation and care? (Select all that apply)	◦ Family members ◦ Friends or neighbors ◦ Online communities ◦ Professional healthcare providers ◦ I have no one to rely on. ◦ I choose not to rely on anyone.

### Interactional dimension: health-related interactions and spirituality

Theoretically, the interactional dimension drew upon the notions that culture and attitudes play a significant role in health inequalities, that doctors’ authority is a form of symbolic capital due to “the positions they occupy in institutionalized healthcare” (1), that the presence of symptoms itself is not necessarily sufficient to motivate health-related interactions (15), and that the experience of healthcare services and products often is mismatched with the underlying intentionality (2). The secondary data further suggested that fear of testing procedures presents a major barrier to participation in prevention initiatives (18).

The expert focus groups discussed that inaccurate beliefs, often formed through online information and/or peers, are a major source of frustration for individual healthcare professionals and a resource-utilization challenge at the systemic level. The 12-CDT data seemed to contradict this, as “Access to digital technologies” was ranked as one of the lowest health-related priorities, indicating a mismatch between individual perceptions underlying self-rated prioritization and the prevailing expert perspectives of healthcare professionals.

Integrated, the sources jointly suggested topics such as healthcare, doctors, prevention, symptoms, experience, fear, beliefs, culture, and attitudes as constituent elements of the interactional dimension. The iterative synthesis of questionnaire items for this dimension yielded a total of seven pairs of questions and answer options, which are presented in the refined version in Table 5.

**Table 5:** Questionnaire items synthesized for the interactional dimension of health capital.

Questionnaire item	Answer options
What might make you delay or avoid seeing a doctor? (Select all that apply)	◦ It is too expensive. ◦ I cannot afford to take time off work. ◦ It is too hard to get there. ◦ I am afraid of the results of the visit. ◦ I have had bad experiences. ◦ Other _______________.
Have you ever felt uncomfortable seeing a doctor due to your… (Select all that apply)	◦ …sex? ◦ …age? ◦ …alcohol use? ◦ …smoking habits? ◦ …body weight? ◦ …sexual orientation? ◦ …culture? ◦ …religion? ◦ …language? ◦ …financial status? ◦ …race or ethnicity? ◦ Other ____________
How often do you or your family participate in health checks or prevention programs such as screening or vaccination?	◦ Never ◦ Rarely ◦ Sometimes ◦ Often ◦ Always
Last time you did not participate in a health check or prevention program, what were the reasons? (Select all that apply)	◦ It was too expensive. ◦ I did not have the time. ◦ I was not interested. ◦ It was not available nearby. ◦ I was afraid of the results or side effects. ◦ Other _______________ ◦ Not applicable
Do you consider yourself religious or spiritual?	◦ Definitely yes ◦ Probably yes ◦ Unsure ◦ Probably not ◦ Definitely not
Would you follow medical advice offered at a religious or cultural event?	◦ Definitely yes ◦ Probably yes ◦ Unsure ◦ Probably not ◦ Definitely not
Does your religious background… (Select all that apply)	◦ …limit which doctors or treatments you use? ◦ …encourage herbal or traditional remedies? ◦ …affect your attitude toward vaccination? ◦ …influence your health choices in other ways? ◦ …not affect your health behavior?

### Behavioral dimension: lifestyle, well-being, and workplace impact

Theoretically, the behavioral dimension drew upon the notions that lifestyle and everyday health-related practices are closely linked, that physical activity often carries symbolic connotations and serves as a means of achieving status and recognition, that economic capital is often perceived as an enabler of positive health behaviors (1), and that basic healthcare skills facilitate supportive healthcare behaviors and, ultimately, better health outcomes (15). The secondary data found that prevention activities organized by employers are often perceived as motivating and suggested that behavioral factors are key determinants of subjective well-being (18).

The expert focus groups discussed how long working hours, mental load, and insufficient sleep affect overall well-being. The 12-CDT data confirmed a link between economic resources and a healthy lifestyle and suggested that health behaviors are linked to mental well-being through “How well I can handle stress,” which represents one of the consistently top-ranked health-related resources.

Integrated, the sources jointly suggested topics such as lifestyle, practices, health, workplace, attitudes, and skills as constituent elements of the behavioral dimension. The iterative synthesis of questionnaire items for this dimension yielded a total of 14 pairs of questions and answer options, which are presented in the refined version in Table 6.

**Table 6:** Questionnaire items synthesized for the behavioral dimension of health capital.

Questionnaire item	Answer options
What roles does physical activity play in your life? (Select all that apply)	◦ It is good for my body. ◦ It is good for my mind. ◦ It gets me out of the house. ◦ It lets me spend time with others. ◦ No role.
Does your health matter when choosing what you eat?	◦ Yes, my health is my main consideration in all food choices. ◦ Yes, my health is one of my main considerations. ◦ I consider my health occasionally, but other factors such as taste, cost, and convenience are often more important. ◦ No, my health does not influence my food choices at all.
What keeps you from eating healthy foods? (Select all that apply)	◦ Healthy foods are too expensive. ◦ I do not have enough time for healthy meals, or they are hard to find. ◦ I am not sure what foods are healthiest or how to prepare them. ◦ I do not like most healthy foods. ◦ Healthy foods do not fit with my culture, traditions, or health needs. ◦ Unhealthy or comfort foods are more convenient. ◦ Other _______________
How often do you consume alcohol?	◦ At least once a day ◦ A few times a week ◦ Once every few months ◦ Never
Does alcohol positively impact your social life, health, or well-being?	◦ Definitely yes ◦ Probably yes ◦ Unsure ◦ Probably not ◦ Definitely not ◦ Not applicable
How often do you consume recreational drugs?	◦ At least once a week ◦ A few times a week ◦ Once every few months ◦ Never
How often do you experience disrupted sleep?	◦ At least once a week ◦ A few times a week ◦ Once every few months ◦ Never
How do you manage disrupted sleep?	◦ Change the physical environment (block noise or light). ◦ Give myself more time to sleep. ◦ Medicine ◦ I cannot do anything about it. ◦ Not Applicable
Are you confident in your ability to manage stress?	◦ Definitely yes ◦ Probably yes ◦ Unsure ◦ Probably not ◦ Definitely not ◦ I never feel stressed.
What challenges at work make it difficult to focus on your health? (Select all that apply)	◦ Long working hours ◦ Stressful workload ◦ Lack of time for breaks ◦ Insufficient managerial support (health and wellness programs) ◦ Other _______________
Does your employer offer health and wellness programs, and if so, do you participate?	◦ Yes, and I participate regularly. ◦ Yes, but I rarely or never participate. ◦ No, my employer does not offer such programs. ◦ I’m unsure if such programs exist.
Does your workplace support your healthcare needs, for example, through health benefits?	◦ Never ◦ Rarely ◦ Sometimes ◦ Often ◦ Always
What do you do to feel better and take care of yourself? (Select all that apply)	◦ Spend time with friends and family ◦ Do hobbies or join group activities ◦ Use online platforms or social media ◦ Take time for myself ◦ Other _______________
What keeps you healthy and feeling well? (Select all that apply)	◦ Being physically active ◦ Feeling calm and not stressed ◦ Supportive family relationships ◦ Friends and partners ◦ Work–life balance ◦ Financial stability ◦ Doing things I enjoy

### Results of the questionnaire mapping

The pre-test phase allowed us to assess completion times and gather feedback on the clarity and sequencing of the questionnaire items. Completion times during pre-testing ranged between 12 and 32 min, with an initial average of 22 min decreasing to just over 16 min following respondent feedback on item comprehensibility, item grouping, and intra- and inter-group flow. Insights from this process informed Phase 3 and its revisions to the questionnaire structure, leading us to reorganize and cluster items into sections that were more intuitive for participants and better aligned with the cognitive world models of the target populations. As an example, we introduced a section “Where and how you live,” which contained questions addressing both demographic and social dimensions of health capital.

The final sections of the questionnaire are presented in Table 7. In addition to the section headings and brief introductions, the remaining columns indicate the number of questions from each of the six dimensions of health capital allocated to each section.

**Table 7:** Sections of the HCQ and the origin of their questionnaire items.

Section heading	Section introduction	Demographic	Economic	Cultural	Social	Interactional	Behavioral	Total
About you	First, we would like to collect some basic information about you.	6	–	2	–	–	–	8
Where and how you live	Now, we would like to know about your home and who you live with.	4	2	–	5	–	–	11
Your interactions with the healthcare system	We would also like to know about your interactions with the healthcare system and where you get health information from.	–	4	5	–	2	–	11
Your attitude toward illness prevention	We are interested in how you and your surroundings view illness prevention.	–	–	2	1	2	–	5
What matters for your health	We want to know more about your everyday life and how it might affect your health.	1	–	–	1	3	12	17
Your well-being	Finally, we are interested in how you and those around you support your well-being.	–	–	–	2	–	2	4
Total		11	6	9	9	7	14	56

## Discussion

The HCQ developed through our three-phase development process is, in many ways, the product of our sources, which, except for the theoretical foundation, consisted of secondary and primary sources of evidence from studies embedded in the Cancer Prevention at Work project (12–14, 18, 21). As such, this concrete HCQ appears well suited to uncover and address sociocultural and behavioral aspects of health in general and barriers to and facilitators of prevention initiatives in similar contexts in particular.

### Strengths and limitations

The HCQ captures, among others, demographic, social, and cultural aspects of health-related personal resources. Some of these are reflected in variables assessed by large-scale instruments on the social determinants of health (17, 29), such as the AHRQ Social Determinants of Health Updated Database (30) with its more than 300 variables. However, these instruments fail to account for personal resources such as household financial and health-related decision-making, social, cultural, and religious influence, and everyday health practices, which have little or no direct analog in, for example, the AHRQ.

The HCQ also differs fundamentally from instruments such as the Health Literacy Questionnaire (HLQ) (31) in both conceptual scope and developmental logic; therefore, the two cannot be directly compared. Although the HLQ is designed to measure the single construct of health literacy, the HCQ captures the broader, inherently multidimensional concept of health capital, whose six categories of dimensions are analytically distinguished but inherently interrelated and, in line with Bourdieusian theory, allow resources in one dimension to be converted into resources in another dimension. Accordingly, although the HLQ focuses on individuals’ capacities to find, understand, evaluate, and apply health information, it also captures personal resources such as household financial and health decision-making, perceived affordability constraints, cultural and religious influences, experiences of discrimination, and health-related everyday practices. Furthermore, HLQ adaptations, such as the Danish version of the HLQ (32), require renewed psychometric validation for each new cultural context, whereas the HCQ, where cultural adaptation and external validation are disentangled, requires external validation against one or more concrete endpoints.

The HCQ is a native, multi-cultural, multi-lingual questionnaire, as its development was embedded in a large-scale European research project, where four culturally and linguistically adapted versions were planned and designed from the outset. That said, the current HCQ may still reflect certain European cultural assumptions and, therefore, may require further adaptation for use in other cultural contexts. In total, two additional adaptations are currently in progress: One for a quite different European context and another for a Global South context. Furthermore, the HCQ has been designed to be administered in different modes, whether using pen and paper, as an online survey, or even as a telephone interview. Similarly, the planned modes of administration include self-administration by respondents and administration by professionals or trained staff.

The 12-CDT represents the most robust source of primary evidence for our study. It has been designed to minimize a range of response biases, such as social desirability bias, through self-administration, and extreme response bias and cognitive load bias, through the use of non-numerical scales (21). Other response biases, such as non-response bias and sampling bias, might still affect the generalizability of the data beyond the populations and settings represented. The 12-CDT is further inherently subject to framing bias, as we purposively preconditioned respondents’ answers by providing a fixed, preselected set of items to reflect on and rank. This aligns with our aim of operationalizing the health capital concept, which adopts a more sociological than psychological lens, and of uncovering and understanding respondents’ health priorities (or “health biases”).

### Methodology

While inspired by other questionnaire development efforts based on conceptual frameworks (10) and qualitative pre-test interviews (33), our approach was based on a more extensive, large-scale mixed-method design, which may provide a stronger basis for its validation-by-design logic.

However, we also acknowledge that the validation-by-design logic does not imply psychometric validity and, therefore, does not remove the need for it. That said, the HCQ exemplifies the advantages of a two-step approach to measuring sociocultural and behavioral phenomena. Instead of presupposing scoring into one or multiple numeric values, the HCQ provides observable data that can be directly employed for correlational and predictive analyses within and across the multiple dimensions of health capital, for example, mapping the impact of different forms of social and cultural capital on health-related behaviors in our study population. Furthermore, these observable data may provide information that can be used to answer different research questions with different endpoints by developing endpoint-specific numerical scoring without having to develop endpoint-specific instruments.

As a hypothetical example, if the endpoint were participation in HPV vaccination, the second step would consist of selecting HCQ items likely to be associated with this endpoint and combining them into an endpoint-specific score. Such a score might, for example, draw on items relating to economic constraints, trust in vaccines and healthcare, access to and understanding of health information, social and religious influence, and prior preventive health practices. The psychometric evaluation of this endpoint-specific score might be conducted in the conventional way in a development sample and tested in an independent sample.

### Directions for future development

The two main directions for the future development of the HCQ are related to external validation and more radical cultural adaptation. Future studies with a predefined endpoint might determine measures for reducing the observable data collected via the HCQ to scores amenable to external validation via exploratory and/or confirmatory factor analysis. To minimize respondent burden, such targeted applications of the HCQ might involve selecting a viable, parsimonious subset of the full HCQ.

Extending the existing adaptations to four European cultural and linguistic contexts beyond English, researchers interested in studying phenomena in the context of the Global South might culturally adapt the HCQ by systematically investigating which questionnaire items need to be added, updated, or deleted to faithfully capture the six dimensions of health capital. Such research would help investigate the extent to which the HCQ might function not as a region-specific questionnaire-based instrument but as a globally applicable and adaptable instrumental framework capable of capturing the socio-cultural dimensions of health across diverse settings.

### Concluding remarks

This article contributes methodologically to instrument development in public health by proposing, implementing, and empirically evaluating a convergent mixed methods approach to questionnaire development for conceptual frameworks. This approach yields a questionnaire-based instrument designed to collect observable data without presupposing a single concrete measurement, thereby providing the opportunity to analyze these data for multiple endpoints and differing constructs.

Furthermore, this article contributes to the assessment of sociocultural and behavioral aspects of public health by developing and making available a unique, adaptable, flexible, and extensible questionnaire-based instrument for assessing health-related resources, based on health capital as an integrated conceptual framework that bridges multiple dimensions.

## Data Availability

The original contributions presented in the study are included in the article/Supplementary material, further inquiries can be directed to the corresponding author.
