# Supplementary material for: Three-phase development of the health capital questionnaire: a mixed methods operationalization study
Source: Front Public Health. 2026 May 18;14:1829250. doi: 10.3389/fpubh.2026.1829250 (PMC13223150; doi:10.3389/fpubh.2026.1829250)
Supplement: Supplementary file 1 [file Presentation_1.pdf]

CPW
CANCER PREVENTION at WORK

SDU
University of Southern Denmark

Welcome to the

Cancer Prevention at Work (CPW) Health Capital Questionnaire.

Take your time and answer as honestly as you can - there are no right or wrong answers. Your answers are important and will help us understand how different parts of your life influence your health.

SECTION 1 - About you

First, we would like to collect some basic information about you.

1. Participant ID - Insert your ID if you have it

a) ______________________________

2. Sex

- o Male
- o Female
- o Other/Undisclosed

3. Age (years)

- o 18-24
- o 25-34
- o 35-44
- o 45-54
- o 55-64
- o 65 or over
- o Prefer not to answer

4. Height (cm)

a) __________________________________

5. Weight (kg)

a) __________________________________

6. Ethnicity

- o American Indian or Alaska Native. A person having origins in any of the original peoples of North and South America (including Central America), and who maintains tribal affiliation or community attachment

This project has received funding from the European Union under grant agreement n° 101104716.

Funded by the European Union

- o Asian. A person having origins in any of the original peoples of the Far East, Southeast Asia, or the Indian subcontinent, including Cambodia, China, India, Japan, Korea, Malaysia, Pakistan, the Philippine Islands, Thailand, and Vietnam
- o Black or African American. A person having origins in any of the black racial groups of Africa.
- o Hispanic or Latino. A person of Cuban, Mexican, Puerto Rican, South or Central American, or other Spanish culture or origin, regardless of race. The term "Spanish origin" can be used in addition to "Hispanic or Latino.
- o Native Hawaiian or Other Pacific Islander A person having origins in any of the original peoples of Hawaii, Guam, Samoa, of other Pacific Islands
- o White. A person having origins in any of the original peoples of Europe, the Middle East, or North Africa
- o Other, specify ______

7. What describes your background?

- o I was born in this country, and my family has been here for several generations.
- o I was born in this country, but my parents moved here from somewhere else.
- o I was born in a different country, but my family moved to this country when I was a child.
- o I moved to this country as an adult.
- o Prefer not to disclose.

8. Education

- o None
- o Primary
- o Secondary (lower than university)
- o Higher (university or higher)
- o Prefer not to answer

9. How many foreign languages do you speak?

- o I don't speak any foreign language
- o 1
- o 2
- o 3
- o 4
- o 5 or more

SECTION 2 - Where and how you live

Now, we would like to know about your home and who you live with.

1. What type of area do you currently live in?

- o Urban (cities, larger towns)
- o Rural (smaller towns, villages, countryside)

This project has received funding from the European Union under grant agreement n° 101104716.

Funded by the European Union

2. Do you feel safe outside your home in your local area?

- o Definitely yes
- o Probably yes
- o Unsure
- o Probably not
- o Definitely not

3. How long does it take to reach the nearest healthcare facility (family doctor, hospital, clinic) from your home?

- o Less than 30 minutes away
- o 30 minutes to 1 hour
- o More than 1 hour away

4. Marital status

- o Single
- o Married
- o Cohabitating with a partner (not married)
- o Divorced/separated
- o Widowed
- o Prefer not to answer

5. How many adult (18+ years) household members do you have?

a. ______

6. How many of your household members are below 18?

a. ______

7. What is your role in your household when it comes to financial support? (Check all that apply)

- o I am the sole provider.
- o Financial responsibilities are shared.
- o I provide for others.
- o Others provide for me.

8. How often do you worry about being able to afford essentials such as rent, food, and/or utilities?

- o Never
- o Rarely
- o Sometimes
- o Often
- o Always

This project has received funding from the European Union under grant agreement n° 101104716.
Funded by the European Union

9. What is your role in your household when it comes to making decisions about healthy eating? (Check all that apply)

- o I make my own decisions.
- o Decisions are shared.
- o I make decisions for others.
- o Others decide for me.

10. What is your role in your household when it comes to making decisions about physical exercise? (Check all that apply)

- o I make my own decisions.
- o Decisions are shared.
- o I make decisions for others.
- o Others decide for me.

11. What is your role in your household when it comes to making decisions about contact with GP/health professionals? (Check all that apply)

- o I make my own decisions.
- o I make decisions for others.
- o Decisions are shared.
- o Others decide for me.

SECTION 3 - Your interactions with the healthcare system

We would also like to know about your interactions with the healthcare system and where you get health information from.

1. Does the public healthcare system meet your needs when you need medical care?

- o Never
- o Rarely
- o Sometimes
- o Often
- o Always

2. Does your family doctor (general practitioner) meet your needs and expectations during visits?

- o Never
- o Rarely
- o Sometimes
- o Often
- o Always

This project has received funding from the European Union under grant agreement n° 101104716.
Funded by the European Union

3. What might make you delay or avoid going to see a doctor? (Check all that apply)

- o It is too expensive.
- o I cannot afford to take time off work.
- o It is too hard to get there.
- o I am afraid of the results of the visit.
- o I have had bad experiences.
- o Other _______________.

4. Have you ever felt uncomfortable seeing a doctor due to your ... (Check all that apply)

- o ... gender?
- o ... age?
- o ... alcohol use?
- o ... smoking habits?
- o ... body weight?
- o ... sexual orientation?
- o ... culture?
- o ... religion?
- o ... language?
- o ... financial status?
- o ... race or ethnicity?
- o Other ____________

5. Do you understand health information provided by your family doctor or other healthcare professionals (like medical specialists, nurses)?

- o Definitely yes
- o Probably yes
- o Unsure
- o Probably not
- o Definitely not

6. Do you understand health-related information you find on your own (e.g., online or in the media)?

- o Definitely yes
- o Probably yes
- o Unsure
- o Probably not
- o Definitely not

7. From whom/where do you get reliable health-related information? (Check all that apply)

- o My healthcare provider
- o Website, app, and social media
- o Family or friends

This project has received funding from the European Union under grant agreement n° 101104716.
Funded by the European Union

- o Books and journals
- o My religious leader or faith group
- o Other ____________

8. How often do you skip or reduce the use of prescribed medication due to its cost?

- o Never
- o Rarely
- o Sometimes
- o Often
- o Always

9. How often do financial constraints influence your ability to make healthier choices like healthy eating, physical exercise, etc.?

- o Never
- o Rarely
- o Sometimes
- o Often
- o Always

10. Do you trust health-related government guidelines for you and your family?

- o Never
- o Rarely
- o Sometimes
- o Often
- o Alway

11. What would strengthen your trust in government health policies or programs? (Check all that apply)

- o Transparency in planning and decisions.
- o Public involvement in planning and decisions.
- o Regular and consistent communication.
- o Policies based on science and evidence.
- o Other _______________
- o I already fully trust them.

SECTION 4 - Your attitude towards illness prevention

We are interested in how you and your surroundings view illness prevention.

This project has received funding from the European Union under grant agreement n° 101104716.
Funded by the European Union

1. Do people you know usually participate in health checks or prevention programmes like vaccinations, cancer screening, or tests for infections?

- o Never
- o Rarely
- o Sometimes
- o Often
- o Always

2. How often do you or your family participate in health checks or prevention programmes like screening or vaccination?

- o Never
- o Rarely
- o Sometimes
- o Often
- o Always

3. Last time you did not participate in a health check or prevention programme, what were the reasons? (Check all that apply)

- o It was too expensive.
- o I did not have the time.
- o I was not interested.
- o It was not available nearby.
- o I was afraid of the result or side effects.
- o Other _______________
- o Not applicable

4. Do you believe screenings can help to detect and therefore to treat cancer?

- o Definitely yes
- o Probably yes
- o Unsure
- o Probably not
- o Definitely not

5. Do you believe vaccines can effectively prevent cancers caused by infections (like Human Papilloma Virus & cervical cancer, Helicobacter pylori & stomach cancer, Hepatitis C Virus & liver cancer)?

- o Definitely yes
- o Probably yes
- o Unsure
- o Probably not
- o Definitely not

This project has received funding from the European Union under grant agreement n° 101104716.

Funded by the European Union

SECTION 5 - What matters for your health

We want to know more about your everyday life and how it might affect your health.

1. Are there histories of illnesses and health issues in your family that make you worry about your own health?

- o Yes, and it makes me worry about my health.
- o Yes, but I do not worry about it.
- o I am not aware of any such histories.

2. How have the habits of your family made it harder for you to stay healthy? (Check all that apply)

- o Expectations around eating or dieting.
- o Cultural or religious practices.
- o Social habits like drinking, smoking.
- o Not at all.

3. What roles does physical activity play in your life? (Check all that apply)

- o It is good for my body.
- o It is good for my mind.
- o It gets me out of the house.
- o It lets me spend time with others.
- o No role

4. Does your health matter when choosing what you eat?

- o Yes, my health is my main consideration in all food choices.
- o Yes, my health is one of my main considerations.
- o I consider my health occasionally, but other considerations like taste, cost, and convenience are often more important.
- o No, my health does not influence my food choices at all.

5. What keeps you from eating healthy foods? (Check all that apply)

- o Healthy foods are too expensive.
- o I do not have enough time for healthy meals, or they are hard to find.
- o I am not sure what foods are healthiest or how to prepare them.
- o I do not like most healthy foods.
- o Healthy foods do not fit with my culture, traditions, or health needs.
- o Unhealthy or comfort foods are more convenient.
- o Other ______________

This project has received funding from the European Union under grant agreement n° 101104716.
Funded by the European Union

6. How often do you consume alcohol?

- o At least once a day
- o A few times a week
- o Once every few months
- o Never

7. Does alcohol positively impact your social life, health, or well-being?

- o Definitely yes
- o Probably yes
- o Unsure
- o Probably not
- o Definitely not
- o Not applicable

8. How often do you consume recreational drugs?

- o At least once a day
- o A few times a week
- o Once every few months
- o Never

9. How often do you experience disrupted sleep?

- o At least once a week
- o A few times a week
- o Once every few months
- o Never

10. How do you manage disrupted sleep?

- o Change the physical environment (block noise or light).
- o Give myself more time to sleep.
- o Medicine
- o I cannot do anything about it.
- o Not Applicable

11. Are you confident in your ability to manage stress?

- o Definitely yes
- o Probably yes
- o Unsure
- o Probably not
- o Definitely not
- o I never feel stressed.

This project has received funding from the European Union under grant agreement n° 101104716.
Funded by the European Union

12. What challenges at work make it difficult to focus on your health? (Check all that apply)

- o Long working hours
- o Stressful workload
- o Lack of time for breaks
- o Insufficient managerial support (health and wellness programs)
- o Other _______________

13. Does your employer offer health and wellness programs, and if so, do you participate?

- o Yes, and I participate regularly.
- o Yes, but I rarely or never participate.
- o No, my employer does not offer such programs.
- o I'm unsure if such programs exist.

14. Does your workplace support your healthcare needs, for example through healthcare benefits?

- o Never
- o Rarely
- o Sometimes
- o Often
- o Always

15. Do you consider yourself religious or spiritual?

- o Definitely yes
- o Probably yes
- o Unsure
- o Probably not
- o Definitely not

16. Would you follow medical advice offered at a religious or cultural event?

- o Definitely yes
- o Probably yes
- o Unsure
- o Probably not
- o Definitely not

17. Does your religious background… (Check all that apply)

- o … limit which doctors or treatments you use?
- o … encourage herbal or traditional remedies?
- o … affect your attitude toward vaccination?
- o … influence your health choices in other ways?
- o … not affect your health behaviour?

This project has received funding from the European Union under grant agreement n° 101104716.
Funded by the European Union

SECTION 6 - Your well-being

Finally, we are interested in how you and those around you support your well-being.

1. How do your friends, colleagues, and other people you know influence your health decisions, for example, lifestyle, vaccinations, screenings? (Check all that apply)

- o They encourage me to make healthy decisions.
- o They offer me support.
- o They judge me for my health decisions.
- o They have no influence on my decisions.

2. When you are unwell, who in your life do you rely on for support like transportation, care? (Check all that apply)

- o Family members
- o Friends or neighbours
- o Online communities
- o Professional healthcare providers
- o I have no one to rely on
- o I choose not to rely on anyone

3. What do you do to feel better and take care of yourself? (Check all that apply)

- o Spend time with friends and family
- o Do hobbies or join group activities
- o Use online platforms or social media
- o Take time for myself
- o Other _______________

4. What keeps you healthy and feeling well? (Check all that apply)

- o Being physically active
- o Feeling calm and not stressed
- o Supportive family relationships
- o Friends and partners
- o Work-life balance
- o Financial stability
- o Doing things I enjoy

This project has received funding from the European Union under grant agreement n° 101104716.

Funded by the European Union
